# Long-term outcomes of patients with cirrhosis presenting with bleeding gastric varices

**DOI:** 10.1371/journal.pone.0264359

**Published:** 2022-03-15

**Authors:** Manabu Nakazawa, Yukinori Imai, Kayoko Sugawara, Yoshihito Uchida, Yoichi Saitoh, Yohei Fujii, Hiroshi Uchiya, Satsuki Ando, Nobuaki Nakayama, Tomoaki Tomiya, Satoshi Mochida

**Affiliations:** Department of Gastroenterology & Hepatology, Faculty of Medicine, Saitama Medical University, Iruma-gun, Saitama, Japan; Nihon University School of Medicine, JAPAN

## Abstract

**Aim:**

To establish a therapeutic strategy for cirrhosis patients with gastric variceal bleeding.

**Methods:**

The outcomes of 137 patients with bleeding gastric varices were evaluated.

**Results:**

The bleeding source was gastroesophageal varices (GOV) in 86 patients, and gastric fundal varices (FV) in 51 patients. The Child-Turcotte-Pugh classes were A, B, and C in 26, 79, and 32 patients, respectively; 34 patients (24.8%) had hepatocellular carcinoma (HCC), of which 11 also had complicating portal venous tumor thrombosis (PVTT). Patients with GOV were treated by endoscopic variceal ligation or endoscopic injection sclerotherapy (EIS) with ethanolamine oleate, while those with FV were treated by EIS with cyanoacrylate; 29 patients with FV also underwent additional balloon-occluded retrograde transvenous obliteration (BRTO). Hemostasis was successfully achieved in 136 patients (99.3%), and the cumulative 1-year, 3-year, and 5-year rebleeding rates were 18.1%, 30.8%, and 30.8%, respectively, in the patients with GOV, and 2.2%, 12.5% and 12.5%, respectively, in the patients with FV. The overall 1-year, 3-year, and 5-year survival rates were 79.7%, 71.5% and 64.4%, respectively, in the patients with GOV, and 91.0%, 76.9% and 59.5%, respectively, in the patients with FV. Multivariable analysis identified PVTT and alcoholic cirrhosis as a significant risk factor associated with rebleeding, model for end-stage liver disease (MELD) score and PVTT as significant factors associated with survival.

**Conclusions:**

Endoscopic therapies with or without BRTO appeared to be useful therapeutic strategies to prevent rebleeding in patients with gastric variceal bleeding, and favorable outcomes were obtained, except in patients with underlying HCC associated with PVTT and/or severe liver damage.

## Introduction

Bleeding varices is one of the major causes of death in patients with liver cirrhosis, and emergency intensive care is often required to rescue these patients [1]. Variceal bleeding may trigger the development of various complications, including renal failure and aggravation of liver damage, and the reported in-hospital mortality rate of cirrhosis patients with variceal bleeding ranges from 15% to 43% [2, 3]. Varices can develop at any site along the gastrointestinal tract in patients with portal hypertension, but gastric varices are encountered less frequently than esophageal varices, and are present in about 20% of patients with cirrhosis [4]. Appropriate management of gastric varices is, however, crucial to obtain favorable outcomes in patients with cirrhosis, as these may cause life-threatening bleeding; bleeding from gastric varices is usually more severe than that from esophageal varices, even if gastric variceal bleeding is less common than esophageal variceal bleeding [4].

Gastric varices are classified into two types: gastroesophageal varices (GOV) and isolated gastric varices (IGV). GOV extend beyond the gastroesophageal junction, connecting with esophageal varices. According to the classification by Sarin *et al*. [5], GOV are further classified into type-1 GOV (GOV-1), located in the lesser curvature, and type-2 GOV (GOV-2), located in the greater curvature, while IGV are classified into type-1 IGV (IGV-1), located in the gastric fundus and/or cardia, and type-2 IGV (IGV-2), located in the pylorus [5]. According to the classification by the Japan Society for Portal Hypertension [6], Lg-c gastric varices correspond to GOV-1 and GOV-2, and Lg-cf or Lg-f gastric varices correspond to IGV-1; the latter are also referred to as gastric fundal varices (FV).

Among the various types of gastric varices, variceal bleeding from FV has been shown to be associated with an especially high mortality rate [5]. Endoscopic variceal ligation (EVL) and endoscopic injection sclerotherapy (EIS) using ethanolamine oleate (EO) are not effective for the initial hemostasis in patients with FV, since these varices, in general, show extremely high blood flows. Therefore, EIS with cyanoacrylate (CA) has been used for the initial hemostasis in patients with FV [7]. However, FV frequently recur, leading to rebleeding, even if initial hemostasis has been successfully achieved by EIS with CA, as long as the blood supply from the gastrorenal shunt (GRS) remains [8, 9]. In contrast, balloon-occluded retrograde transvenous obliteration (BRTO), which has been used in Japan as elective therapy for patients with FV showing blood supply through GRS, has seldom been shown to be followed by recurrence of FV bleeding [10–13]. Therapeutic strategy for gastric variceal bleeding has not yet been established. Usefulness of endoscopic therapies and that of BRTO were shown in the clinical practice guideline for liver cirrhosis in Japan [14, 15], while the indication of each therapy and the significance of combined BRTO plus endoscopic therapies were to be clarified in the future. Thus, at our institution, we undertake endoscopic therapies with or without BRTO for patients with gastric variceal bleeding, depending on the types and hemodynamics of the varices. In the present paper, the usefulness of our therapeutic strategy was evaluated based on the long-term outcomes of patients presenting with gastric variceal bleeding.

## Patients and methods

### Patients

The subjects were 137 patients with bleeding gastric varices in whom measures for the initial hemostasis were undertaken at Saitama Medical University Hospital between January 2006 and July 2020. Bleeding from gastric varices was diagnosed when endoscopic examinations in patients presenting with upper gastrointestinal bleeding symptoms revealed active bleeding from varices and/or fibrin clots, erosions or ulcers on the surfaces of varices.

The types of gastric varices were classified according to the classification of the Japan Society for Portal Hypertension [6], and patients having Lg-c gastric varices were classified as those with GOV and those having Lg-cf or Lg-f gastric varices were classified as those with FV. The extents of liver damage were assessed according to the Child-Turcotte-Pugh classification, model for end-stage liver disease (MELD) score, albumin-bilirubin (ALBI) score, and the modified ALBI (mALBI) grades [16]. The long-term outcome of patients, including the rebleeding rates and survival rates, were examined retrospectively. Bleeding from both esophageal varices and gastric varices during the follow-up period following the initial hemostasis for gastric varices was counted as rebleeding.

Written informed consent for endoscopic procedures and BRTO were obtained from all the patients. This retrospective study was reviewed and approved by an institutional review board of Saitama Medical University Hospital before the study began (20151.01).

### Therapeutic strategy

Patients with bleeding GOV received EVL or EIS using EO for the initial hemostasis. EO was injected through the lumen of the esophageal varices and the arrival of the EO into the feeding vessels was confirmed by x-ray fluoroscopy. EVL, EIS with EO, and argon plasma coagulation (APC) were adopted as additional therapies in these patients, depending on the therapeutic efficacy of the initial hemostasis procedure and the size and hemodynamics of the varices at the baseline. In contrast, patients with bleeding FV underwent EIS using CA for the initial hemostasis. In patients seen until November 2014, 75% α-cyanoacrylate monomer was used, while in those seen in December 2014 and later, 75% n-butyl-2-cyanoacrylate was used. BRTO was performed as an additional therapy in patients with GRS, while EIS using both CA and EO was performed in patients without the shunts. Patients in whom both the feeding vessels and the FV were found, on CT, to be completely occluded following EIS with CA were not treated by additional BRTO. Furthermore, patients with Child-Turcotte-Pugh class C liver damage received additional BRTO following improvement of the liver damage to at least Child-Pugh class B. Endoscopic examinations were done at first from 3 to 6 months following the discharge, and then after, the examinations were done every 6 to 12 months. The survival periods were calculated based on the duration from gastric variceal bleeding onset to death or the recent clinical visits.

### Statistical analysis

The chi-squared test or Fisher’s exact test was used for statistical comparison of the variables. The cumulative survival rates and cumulative recurrence rates of variceal bleeding were evaluated by the Kaplan-Meier method, followed by comparison by the log-rank test. Multivariable regression analysis using a Cox’s proportional hazards model was used to identify factors associated with the survival and the risk factors for variceal rebleeding using factors which were identified as statistical significance in the univariate analysis. *P* values of less than 0.05 were considered as denoting statistical significance.

## Results

### Demographic features and clinical characteristics of the patients with gastric variceal bleeding

The demographic features and clinical characteristics of the 137 enrolled patients are shown in Table 1. The patients consisted of 88 men (64.2%) and 49 women (35.8%), with a median age of 65 years (range, 30 to 91 years). Of the 137, 86 patients (62.8%) had bleeding GOV, while 51 patients (37.2%) had bleeding FV. The Child- Turcotte-Pugh class was A, B, and C in 26 (19.0%), 79 (57.7%), and 32 (23.3%) patients, respectively, and the mALBI grades were 1, 2a, 2b, and 3 in 8 (5.8%), 9 (6.6%), 82 (59.9%), and 38 (27.7%) patients, respectively, and portal venous thrombosis (PVT) was found in 5 patients (3.6%). Esophageal varices were present in 118 patients (86.1%). Of the total, 34 patients (24.8%) had underlying hepatocellular carcinoma (HCC), with or without a history of previous therapies, of whom 11 also had portal venous tumor thrombosis (PVTT); the extents of the PVTT were Vp1 (subsegmentary), Vp2 (secondary order branch), Vp3 (first-order blanch), and Vp4 (main trunk) in 0 (0%), 1 (2.9%), 4 (11.8%), and 6 (17.6%) patients, respectively. Thirty-four patients consisting of 25 patients with GOV and 9 patients with FV were given proton pomp inhibitors (PPI) prior to gastric variceal bleeding. Patients receiving beta blockers were absent.

**Table 1:** Demographic features and clinical characteristics of the 137 enrolled patients with cirrhosis who presented with bleeding gastric varices.

Characteristics		GOV n = 86	FV n = 51	*P* value
Age : years old	medium (range)	64.5 (30–91)	67.2 (38–85)	0.0558
Sex	men : women	59 : 27	29 : 22	0.1981
Etiology	HCV : HBV : alcohol : others	30 : 2 : 32 : 22	20 : 0 : 16 : 15	0.6072
Child-Turcotte-Pugh class	A : B : C	14 : 50 : 22	12 : 29 : 10	0.4210
MELD score	medium (range)	9 (6–29)	10 (7–27)	0.9204
mALBI grade	1 : 2a : 2b : 3	6 : 4 : 50 : 26	2 : 5 : 32 : 12	0.4867
PVT	absent : present	82 : 4	50 : 1	0.6507
HCC	absent : present	60 : 26	43 : 8	0.0670
PVTT (Vp)	0 : 1 : 2 : 3 : 4	15 : 0 : 1 : 4 : 6	8 : 0 : 0 : 0 : 0	0.0339
Esophageal varices	absent : present	0 : 86	19 : 32	<0.0001
PPI	absent : present	61 : 25	42 : 9	0.1559

GOV: gastroesophageal varices, FV: gastric fundal varices, HCV: hepatitis C virus, HBV: hepatitis B virus, MELD: model for end-stage liver disease, mALBI: modified albumin bilirubin, PVT: portal venous thrombosis, HCC: hepatocellular carcinoma, PVTT; portal venous tumor thrombosis, PPI: proton pump inhibitors.

### The initial hemostasis procedure in the patients who presented with gastric variceal bleeding

**Fig 1:**
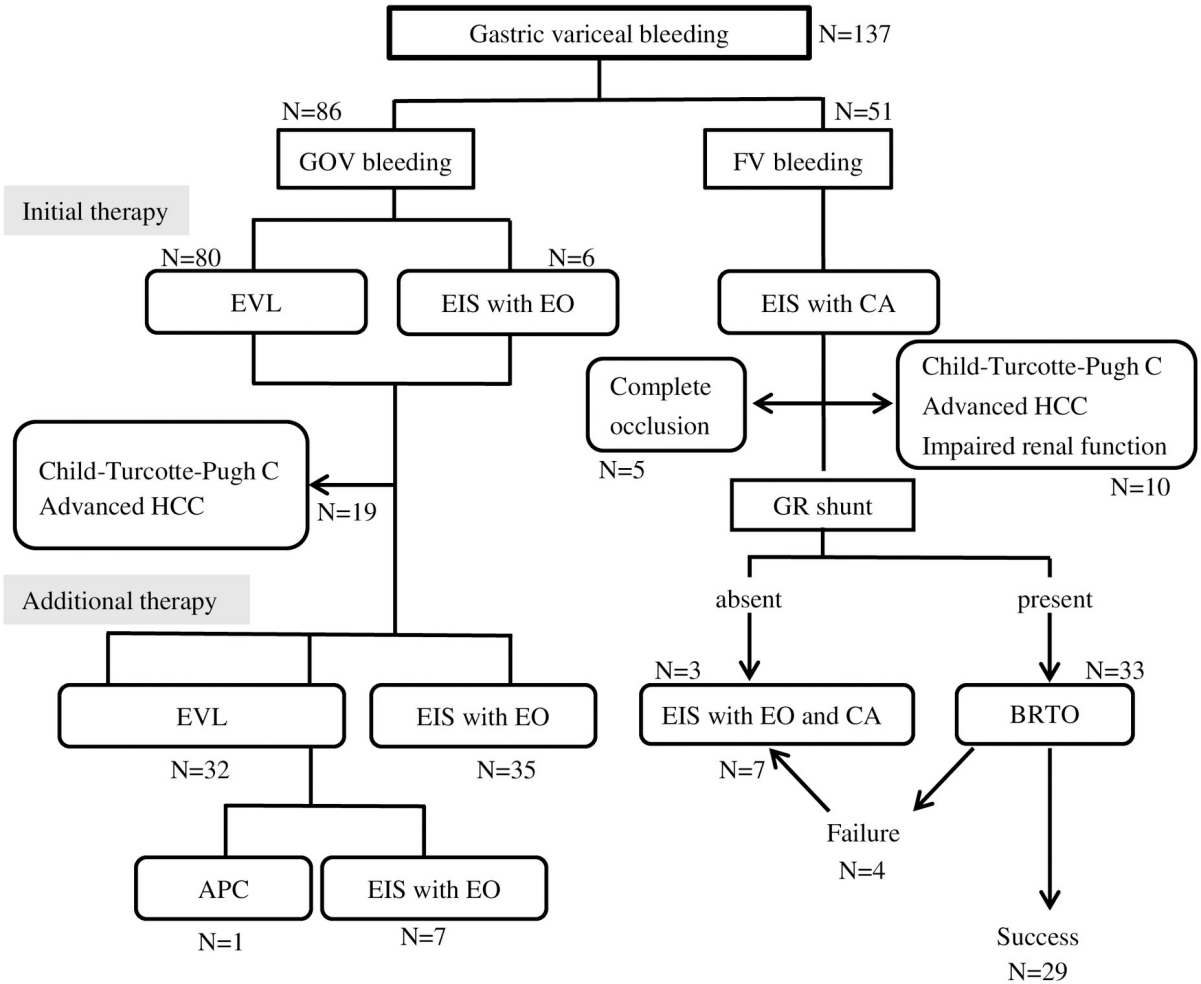
Therapies adopted in the 137 enrolled patients who presented with gastric variceal bleeding. EVL, Endoscopic variceal ligation; EIS, endoscopic injection sclerotherapy; EO, ethanolamine oleate; CA, cyanoacrylate; HCC, hepatocellular carcinoma; CKD, chronic kidney disease; GRS, gastrorenal shunt; BRTO, balloon-occluded retrograde transvenous obliteration; APC, argon plasma coagulation.

Among the 86 patients with bleeding GOV, 80 patients (93.0%) received EVL, and the remaining 6 patients (7.0%) underwent EIS with EO for the initial hemostasis. Among the 51 patients with bleeding FV, 36 patients (70.6%) underwent EIS with the 75% α-cyanoacrylate monomer, and 13 patients (25.5%) underwent EIS with 75% n-butyl-2-cyanoacrylate; 2 patients (3.9%) received both the sclerosing agents at the time of the initial hemostasis (Fig 1).

Initial hemostasis was successfully achieved in 136 (99.3%) of the 137 patients, excluding one patient with bleeding GOV. Eleven patients (8.0%) died during the hospitalization, and the cause of death was HCC progression in 5 patients, liver failure in 3 patients, bacterial infection in 2 patients, and rupture of esophageal varices after EIS with CA for FV in 1 patient.

### Additional therapies for patients with recurrent bleeding gastric varices after successful initial hemostasis

Among the 86 patients with bleeding from GOV, 35 (40.7%), 24 (27.9%),7 (8.1%), and 1 (1.2%) patient(s) received EIS with EO, EVL, EIS with EO following EVL, and APC following EVL, respectively, as additional therapies. The remaining 19 patients (22.1%) did not receive any additional therapies due to the severe underlying liver damage and/or HCC progression. These patients were given conventional supportive care therapies including oral PPI administration following the initial hemostasis, while those receiving beta blockers were absent.

Among the 51 patients with bleeding from FV, 36 patients (70.6%) received additional therapies. On contrast-enhanced CT, GRS were detected in 33 patients (64.7%), while the left inferior phrenic vein was noted as the main drainage vessel in the remaining 3 patients (5.9%). Of the 33 patients with flow from the GRS, BRTO procedures were done successfully in 29 patients (87.9%), while the remaining 4 patients underwent EIS with both EO and CA following failure of the BRTO procedures. The 3 patients without GRS also underwent EIS with both EO and CA. The remaining 15 patients (29.4%) did not receive any additional therapies due to complete occlusion of the shunt vessels after the initial hemostasis (5 patients) or due to severe underlying liver damage (6 patients), HCC progression (2 patients), and/or impaired renal function (1 patient), while 1 patient received additional therapy at another hospital (Fig 1). The patients without receiving the additional therapies were given conventional supportive care therapies including oral PPI administration following the initial hemostasis, while those receiving beta blockers were absent.

### Long-term outcomes of the patients with gastric variceal bleeding

**Fig 2:**
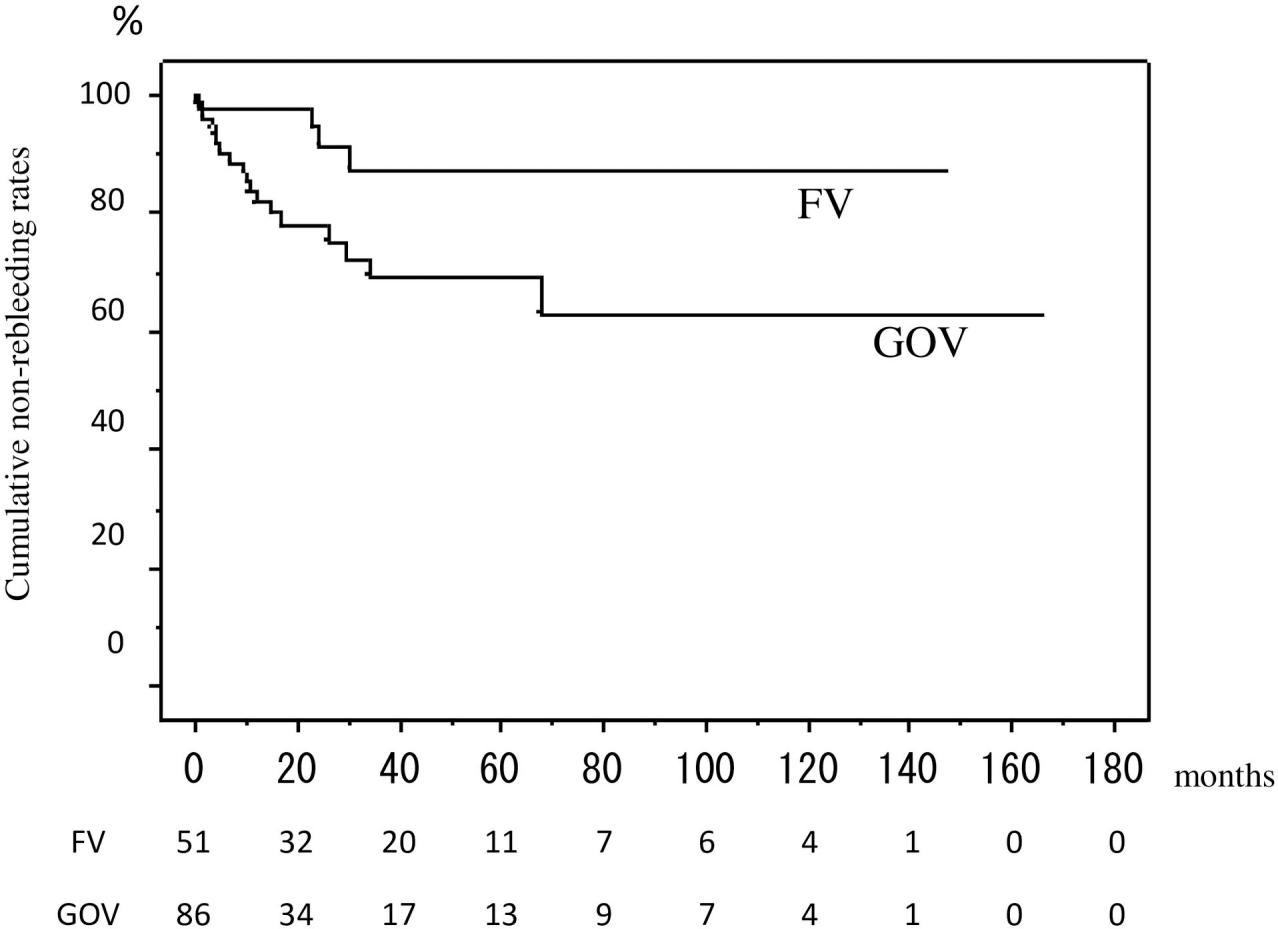
Cumulative rebleeding rates in the 137 enrolled patients who presented with gastric variceal bleeding. The cumulative 1-year, 3-year, and 5-year rebleeding rates were 18.1%, 30.8% and 30.8%, respectively, in the patients with bleeding GOV, and 2.2%, 12.5% and 12.5% in the patients with bleeding FV.

Of the 86 patients with bleeding GOV, rebleeding occurred in 18 patients (20.9%). Of the 18 patients, the rebleeding was from esophageal varices in 6 patients and from the GOV in 12 patients; the cumulative 1-year, 3-year and 5-year rebleeding rates were 18.1%, 30.8% and 30.8%, respectively. In contrast, of the 51 patients with bleeding FV, rebleeding occurred in 4 patients (7.8%). Of the 4 patients, the rebleeding was from esophageal varices in 3 patients, and from GOV in 1 patient; the cumulative 1-year, 3-year and 5-year rebleeding rates were 2.2%, 12.5% and 12.5%, respectively. None of the patients with initial bleeding from either GOV or FV developed rebleeding from FV (Fig 2).

The rebleeding rates were higher in patients with underlying HCC and complicating PVTT than in those without underlying HCC and/or PVTT (P<0.0001); the 1-year rebleeding rate in the former group was 60.5%, while the 1-year, 3-year, and 5-year rebleeding rates in the latter group were 9.2%, 21.2%, and 21.2%, respectively. PVT, however, did not affect the rebleeding rates in these patients. The rates were higher in patients with alcoholic cirrhosis than in those with cirrhosis due to other etiologies (P = 0.0037) (Table 2). Moreover, the rebleeding rates were higher in patients with initial bleeding from GOV than in those with initial bleeding from FV (P = 0.0211). In contrast, the rates were not different between patients receiving PPI prior to the initial gastric variceal rebleeding and the remaining patients. Multivariate analysis, however, identified PVTT and alcoholic cirrhosis as the significant risk factors for rebleeding, with the hazard ratio of 10.421 (P<0.001) and 2.891 (p = 0.0218), respectively, while the initial bleeding sites, either GOV or FV, were not identified.

**Table 2:** Cumulative rebleeding rates of the 137 enrolled patients with gastric variceal bleeding and the risk factors for rebleeding.

		Kaplan Meier method					Cox proportional hazard regression analysis		
		Cumulative Rebleeding rates (%)				P values	HR	95% CI	*P* values
		N	1 year	3 years	5 years				
Total		137	12.1	23.7	23.7				
Age: years	< 65	67	16.1	31.7	31.7	0.0597			
	≥ 65	70	7.5	14.1	14.1				
Etiology	Alcohol	48	22.0	39.6	39.6	0.0037	2.891	1.163–6.836	0.0218
	Others	89	6.8	14.3	14.3		1		
Child-Turcotte-Pugh class	A	26	4.0	10.0	10.0	0.1789			
	B,C	111	14.7	28.0	28.0				
MELD score	< 10	69	10.1	24.3	24.3	0.9427			
	≥ 10	68	14.4	22.2	22.2				
mALBI grade	1, 2a	17	12.5	30.0	30.0	0.7676			
	2b, 3	120	12.1	22.6	22.6				
PVT	absent	132	11.8	23.7	23.7	0.7262			
	present	5	20.0	20.0	20.0				
HCC	absent	103	9.8	24.3	24.3	0.6324			
	present	34	20.2	20.2	20.2				
PVTT (Vp)	absent	126	9.2	21.2	21.2	<0.0001	1	3.060–35.492	0.0002
	present	11	60.5	-	-		10.421		
Esophageal varices	absent	19	0	0	0	-			
	present	118	14.2	28.2	28.2				
Site of bleeding	GOV	86	18.1	30.8	30.8	0.0211	2.581	0.846–7.876	0.0957
	FV	51	2.2	12.5	12.5		1		
PPI	absent	103	8.7	21.8	21.8	0.3238			
	present	34	22.5	29.5	29.5				

N: number of patients, HP: hazard ratio, CI: confidence interval, HCV: hepatitis C virus, HBV: hepatitis B virus, MELD: model for end-stage liver disease, mALBI: modified albumin bilirubin, PVT: portal venous thrombosis, HCC: hepatocellular carcinoma, PVTT (Vp); portal venous tumor thrombosis, GOV: gastroesophageal varices, FV: gastric fundal varices, PPI: Proton pump inhibitors.

**Fig 3:**
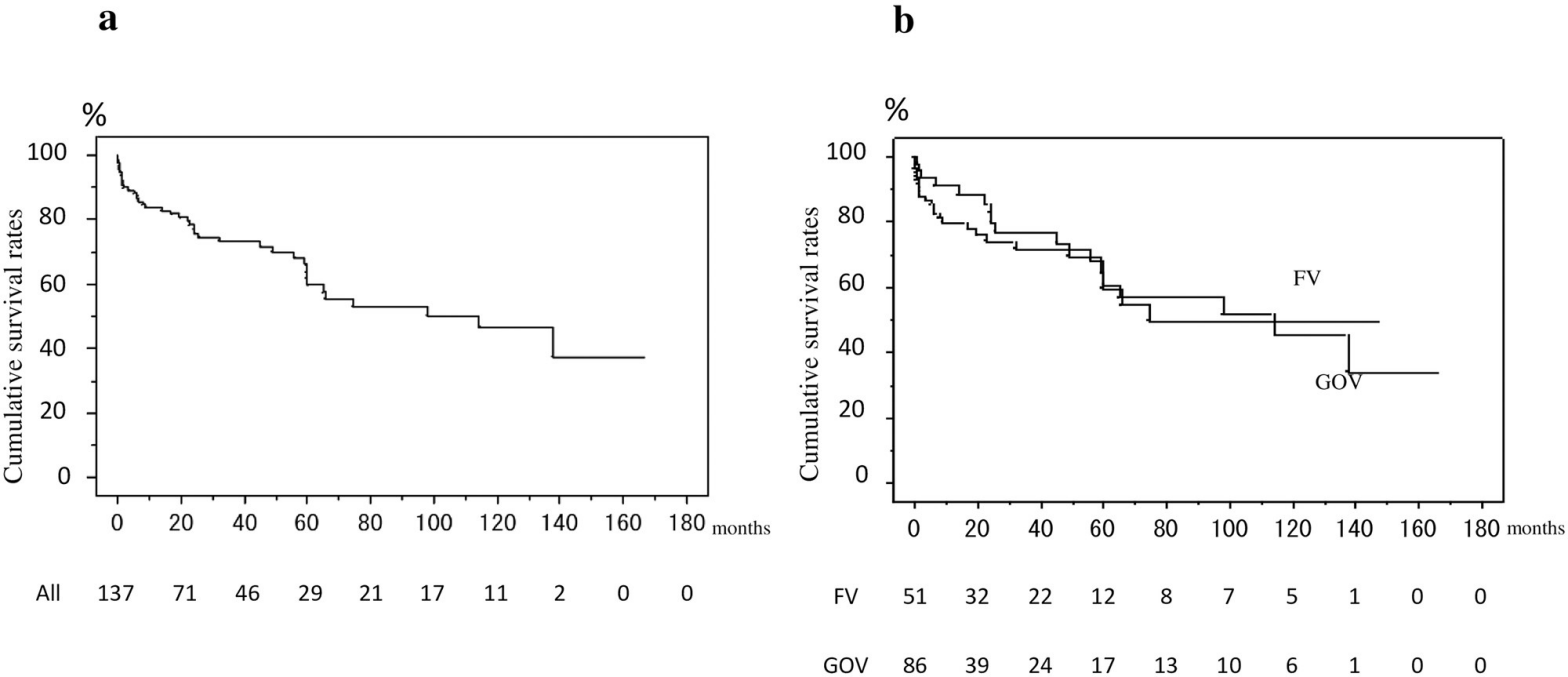
Cumulative survival rates of the 137 enrolled patients who presented with gastric variceal bleeding. a) The 1-year, 3-year, and 5-year overall survival rates were 83.8%, 73.3% and 61.8%, respectively; b) The 1-year, 3-year, and 5-year survival rates were 79.7%, 71.5% and 64.4%, respectively, in the patients with bleeding GOV, and the rates were comparable to those in patients with bleeding FV (91.0%, 76.9%, and 59.5%, respectively).

The 3-year, 3-year, and 5-year overall survival rates of the 137 patients were 83.8%, 73.3% and 61.8%, respectively (Fig 3a); the rates did not differ significantly between the 86 patients with bleeding GOV and 51 patients with bleeding FV; the rates were 79.7%, 71.5% and 64.4%, respectively, in the former group, and 91.0%, 76.9% and 59.5%, respectively, in the latter group (Fig 3b). Thirty-one patients died during the follow up, and the cause of death was liver failure in 12 patients, rupture of esophageal varices in 5 patients, HCC progression in 4 patients, bacterial infection in 3 patients, rupture of GOV in 2 patients and lung cancer, pancreatic cancer, bowel obstruction, traffic accident, unknown cause in 1 patient, respectively.

As shown in Table 3, the 1-year, 3-year, and 5-year survival rates were higher in the Child-Turcotte-Pugh class A patients than in the Child-Turcotte-Pugh class B or C patients (P = 0.0375); the 1-year, 3-year, and 5-year survival rates were 100%, 94.7% and 86.1%, respectively, in the former group, while the corresponding rates were 79.6%, 67.5%, and 54.8% in the latter group. Moreover, the rates were higher in patients with MELD score of <10 than in those with score of ≥10 (p = 0.0002). In contrast, the survival rates were higher in patients without underlying HCC than in those with underlying HCC (P = 0.0014); the 1-year, 3-year, and 5-year survival rates were 91.2%, 78.7%, and 66.9%, respectively, in patients without underlying HCC, while the corresponding rates were 60.7%, 56.7%, and 45.3%, respectively, in those with underlying HCC. The rates were also higher in patients without PVTT, even if there was underlying HCC than in those with underlying HCC with PVTT (P<0.0001); the 1-year, 3-year, and 5-year survival rates were 89.2%, 78.0% and 65.8%, respectively, in the former group, while the 1-year survival rate was 18.2% in the latter group. Moreover, the 1-year, 3-year, and 5-year survival rates were higher in patients without rebleeding than in those who developed rebleeding (P = 0.0198); the rates were 86.5%, 77.9%, and 67.0%, respectively, in the former group, and 72.0%, 55.4% and 42.7%, respectively, in the latter group.

**Table 3:** Cumulative survival rates of the 137 enrolled patients with gastric variceal bleeding and factors associated with the outcomes of the patients.

		Kaplan Meier method					Cox proportional hazard regression analysis		
		Cumulative Survival rates (%)				P values	HR*	95% CI*	*P* values*
		N	1 year	3 years	5 years				
Total		137	83.8	73.3	61.8	−			
Age: years	< 65	67	85.4	76.1	61.1	0.8772			
	≥ 65	70	82.4	70.4	66.0				
Etiology	Alcohol	48	90.4	83.7	65.7	0.6925			
	Others	89	80.3	67.8	61.0				
Child-Turcotte-Pugh class	A	26	100.0	94.7	86.1	0.0375	1	0.716–5.018	0.1982
	B, C	111	79.6	67.5	54.8		1.895	0.632–4.517	0.2957
							1.690		
MELD score	< 10	69	93.6	91.6	81.2	0.0002	1	1.586–6.202	0.0010
	≥ 10	68	73.0	52.8	39.4		3.136	1.692–6.709	0.0005
							3.369		
mALBI grade	1, 2a	17	93.8	93.8	76.7	0.1856			
	2b, 3	120	82.4	70.1	59.7				
PVT	Absent	132	84.0	73.2	61.4	0.7749			
	present	5	80.0	80.0	80.0				
HCC	absent	103	91.2	78.7	66.9	0.0014	1	0.451–2.477	0.8990
	present	34	60.7	56.7	45.3		1.057	0.503–2.873	0.6791
							1.202		
PVTT (Vp)	absent	126	89.2	78.0	65.8	< 0.0001	1	5.683–63.365	< 0.0001
	present	11	18.2	-	-		18.977	3.951–49.212	< 0.0001
							13.945		
Esophageal varices	absent	19	100.0	92.3	92.3	0.0735			
	present	118	81.3	70.3	56.6				
Site of bleeding	GOV	86	79.7	71.5	64.4	0.5535			
	FV	51	91.0	76.9	59.5				
PPI	absent	103	86.5	77.9	67.0	0.6789			
	present	34	78.6	74.3	74.3				
Rebleeding	absent	115	86.5	77.9	67.0	0.0198	1		
	present	22	72.0	55.4	42.7		-	-	-
							1.907	0.948–3.838	0.0701

*upper and lower values indicate those obtained from analysis with factors at baseline and those from analysis with factors including rebleeding following initial hemostasis.

N: number of patients, HP: hazard ratio, CI: confidence interval, HCV: hepatitis C virus, HBV: hepatitis B virus, MELD: model for end-stage liver disease, mALBI: modified albumin bilirubin, PVT: portal venous thrombosis, HCC: hepatocellular carcinoma, PVTT (Vp); portal venous tumor thrombosis, GOV: gastroesophageal varices, FV: gastric fundal varices, PPI: Proton pump inhibitors.

Multivariate analysis including factors assessed at the baseline identified the MELD score and PVTT as significant factors associated with the survival, with hazard ratios of 3.136 and 18.977, respectively (P = 0.001 and P<0.0001, respectively). The analysis based on additional factors, including rebleeding, also identified MELD score and PVTT as the significant factors associated with the survival, with a hazard ratio of 3.369 and 13.945, respectively (P = 0.0005 and P<0.0001, respectively).

## Discussion

In the present study, the usefulness of endoscopic therapies with/without BRTO was evaluated in 137 patients who presented with gastric variceal bleeding. The patients received EVL, EIS with EO, or EIS with CA as the initial hemostasis procedure depending on the types and sizes of the gastric varices, and favorable short-term outcomes were obtained; the overall hemostasis rate and in-hospital mortality rate were 99.3% and 8.0%, respectively, after the initial hemostasis procedure. Favorable short-term outcomes were obtained even in patients showing bleeding from FV, in which the blood flows are, in general, extremely high; the hemostasis rate in this patient was 100%, equivalent to that in patients showing bleeding from GOV (98.8%). Recently, Sato M, *et al*. reported based on the nationwide database in Japan that the in-hospital mortality rate of 9,987 patients with bleeding esophageal or gastric varices was 16% [3]. Notwithstanding previous reports that the short-term outcomes of patients with bleeding gastric varices is unfavorable in comparison with that of patients with bleeding esophageal varices, the in-hospital mortality rate at our institute was more than 2 times lower than that reported from other hospitals in Japan, suggesting that our strategy for the initial hemostasis contributed to successful achievement of hemostasis in almost all patients, with a resultant reduction of the in-hospital mortality rates.

Esophageal varices are present in all patients with bleeding GOV, suggesting that aggravation of esophageal varices as well as GOV recurrence are inevitable in these patients, even if the initial hemostasis was successfully achieved. Thus, in the 86 patients with bleeding from GOV, EVL, EIS with EO, and APC were performed, either alone or in combination, as additional therapies depending on the therapeutic efficacy of the initial hemostasis procedure and the sizes and hemodynamics of the GOV at the baseline, to prevent rebleeding. However, rebleeding did occur from esophageal varices in 6 patients and GOV in 12 patients. The cumulative 1-year, 3-year, and 5-year rebleeding rates were 18.1%, 30.8% and 30.8%, respectively. The rebleeding rates in the present study were lower than those reported for patients who had undergone EVL for gastric variceal bleeding and almost similar to those reported for patients who had undergone EIS using CA [17, 18]. Tan PC *et al*. reported that the cumulative 1-year, 2-year, 3-year, and 4-year rebleeding rates in patients showing gastric variceal bleeding (from GOV in 80% and from FV in 20%) were 33.5%, 63.1%, 72.3% and 81.6%, respectively, after the initial hemostasis by EVL, and 22.8%, 26.8%, 26.8% and 26.8%, respectively, after the initial hemostasis by EIS using CA [17]. Moreover, Lo GH *et al*. reported that rebleeding occurred in 54% of patients after EVL and 31% of patients after EIS using CA in patients with gastric variceal bleeding (from GOV in 92% and from FV in 8%) [18]. These observations suggest that the therapeutic strategy for patients showing bleeding from GOV, of using additional therapies, except for Child-Turcotte-Pugh class C patients and/or those with advanced HCC, was superior to the conventional therapeutic strategy of EVL alone, and yielded a similar efficacy to that of EIS using CA. In the present study, no patients received non-selective beta blockers, which were recommended for patients showing variceal bleeding to prevent rebleeding in the Baveno VI consensus workshop and the American Association for the Study of Liver Diseases (AASLD) guideline [4, 7]. Usefulness of non-selective beta blockers should be also investigated in these patients. Moreover, the bleeding rates were higher in patients with alcoholic cirrhosis than in those with cirrhosis due to the other etiologies. Previously, Nakamura *et al*. reported that esophageal varices were frequently found in cirrhotic patients with non-alcoholic steatohepatitis (NASH) as well as alcoholic liver diseases [19]. The significance of steatohepatitis in the rebleeding should be investigated in future, since patients with NASH, which were diagnosed on histological examinations were not included in the present study.

In contrast, the 51 patients with bleeding FV underwent BRTO and/or EIS with both EO and CA as additional therapies following the initial hemostasis procedure of EIS using CA, to occlude the feeding vessels. The aggravation of esophageal varices was shown to develop frequently in patients with gastric fundal varices following BRTO procedures [12, 13, 20]. Thus, in the present study, the rebleeding rates were also evaluated in these patients. Consequently, none of the patients developed rebleeding from the FV, and the 1-year, 3-year, and 5-year cumulative rebleeding rates from esophageal varices/GOV were 2.2%, 12.5% and 12.5%, respectively. The long-term outcomes of patients with bleeding FV were markedly favorable in comparison with those reported from a previous study; the cumulative 1-year, 5-year, and 10-year rebleeding rates were 36.3%, 47.3%, and 51.8%, respectively, following EIS using CA in patients with bleeding FV, according to a report by Akahoshi T, *et al* [8]. Thus, the therapeutic strategy for patients with bleeding FV, of using additional therapies such as BRTO or EIS with both EO and CA, was useful for preventing rebleeding from esophageal and/or gastric varices in patients with bleeding FV, after the initial hemostasis procedure of EIS using CA. According to the 2016 practice guideline published by AASLD, transjugular intrahepatic portosystemic shunt (TIPS) or BRTO is recommended as the first-line therapy to prevent rebleeding in patients with bleeding FV [4]. BRTO is reported to be superior to TIPS for preventing rebleeding after the initial hemostasis procedure for gastric variceal bleeding [21, 22], although no prospective trial comparing the efficacies of BRTO and TIPS has been reported yet. In the present study, EIS using both EO and CA was performed as additional therapy after the initial hemostasis by EIS using CA in patients without GRS and those with failure of the BRTO procedure. The usefulness of EIS using both EO and CA should be investigated in comparison with that of TIPS as additional therapy to EIS using CA in the future.

In the present study, the overall 1-year, 3-year, and 5-year survival rates after the initial hemostasis in the patients with gastric variceal bleeding were 83.8%, 73.3% and 61.8%, respectively, and the rates did not differ significantly between patients with bleeding GOV and bleeding FV. In contrast, Tan PC *et al*. reported that the 1-year and 3-year overall survival rates after the initial hemostasis obtained with EVL in the patients with gastric variceal bleeding were 55.5% and 37.2%, respectively, and the corresponding rates after the initial hemostasis with EIS using CA were 57.6% and 43.1%, respectively [17]. Thus, endoscopic therapies and BRTO were useful therapeutic strategies to improve the survival rates, as well as reduce the rebleeding rates in patients with gastric variceal bleeding. There were limitations, however, of the therapeutic strategy. As shown in Table 2, multivariate analysis identified PVTT as a significant risk factor associated with rebleeding. Furthermore, as shown in Table 3, the MELD score and PVTT were identified as significant factors affecting the survival. In patients with gastric variceal bleeding who had HCC complicated by PVTT and/or severe liver damage, the long-term outcomes were unfavorable, even if initial hemostasis was achieved successfully. Previously, Komori *et al*. reported that the long-term overall survival rate was significantly lower in patients receiving PPI regularly than in those who did not use PPI regularly [23]. In the present study, however, PPI were not identified as a significant factor associated with the rebleeding rates as well as survival rates in these patients probably due to regular intake of PPI in almost all patients following hemostasis.

In conclusion, Endoscopic therapies and BRTO were useful therapeutic strategies to prevent rebleeding from esophageal and gastric varices, and improved overall survival rates were obtained in the patients with gastric variceal bleeding, except those with HCC and complicating PVTT, and/or patients with severe liver damage.

## Supporting information

S1 File (PDF)

S2 File (PDF)
