# Supplementary material for: Long-term outcomes of patients with cirrhosis presenting with bleeding gastric varices
Source: PLoS One. 2022 Mar 15;17(3):e0264359. doi: 10.1371/journal.pone.0264359 (PMC8923466; doi:10.1371/journal.pone.0264359)
Supplement: S1 File — (PDF) [file pone.0264359.s001.pdf]

sex	ALB	TB	ALBI score	ALBI grade	MELD	出血源	食道静脈瘤有無
m	3.4	0.4	−2.34	2a	7	Lg_c	あり
m	3.9	1	−2.5	2a	10	Lg_c	あり
f	3.2	1.1	−1.88	2b	9	Lg_f	あり
f	2.4	3.1	−0.9	3	16	Lg_f	あり
m	4.2	1	−2.76	1	9	Lg_c	あり
f	3.1	1.5	−1.71	2b	9	Lg_f	あり
m	2.4	2.1	−1.01	3	15	Lg_c	あり
f	3.8	1.3	−2.34	2a	13	Lg_f	あり
m	2.6	1.3	−1.32	3	9	Lg_f	あり
f	3.7	1	−2.33	2a	9	Lg_f	なし
m	2.9	1.1	−1.62	2b	9	Lg_c	あり
f	3.2	1	−1.91	2b	11	Lg_c	あり
m	3.7	1.5	−2.22	2b	9	Lg_c	あり
f	3.4	1.3	−2	2b	8	Lg_f	なし
m	4	0.5	−2.78	1	8	Lg_c	あり
m	3	1	−1.74	2b	9	Lg_c	あり
m	4.4	0.5	−3.12	1	7	Lg_c	あり
f	3.4	0.5	−2.27	2b	6	Lg_c	あり
f	3.5	2.9	−1.86	2b	8	Lg_c	あり
m	2.8	2.1	−1.35	3	10	Lg_f	なし
m	2.7	1.2	−1.43	2b	20	Lg_c	あり
m	2.8	1.2	−1.51	2b	13	Lg_f	なし
m	2.5	1.1	−1.28	3	22	Lg_c	あり
m	3	1.7	−1.58	2b	10	Lg_f	なし
m	2.6	1.9	−1.21	3	24	Lg_c	あり
m	3.7	1.1	−2.3	2a	18	Lg_f	なし
f	3.1	0.9	−1.85	2b	11	Lg_c	あり
f	2.4	3.8	−0.84	3	11	Lg_c	あり
m	3.4	2.4	−1.83	2b	16	Lg_c	あり
m	2.9	1.2	−1.6	2b	20	Lg_c	あり
m	3.3	0.8	−2.06	2b	7	Lg_f	なし
f	3.5	0.7	−2.26	2b	9	Lg_c	あり
f	2.8	0.4	−1.83	2b	7	Lg_c	あり
m	3	0.9	−1.77	2b	9	Lg_c	あり
m	3.1	1.2	−1.77	2b	11	Lg_c	あり
m	2.7	0.7	−1.58	2b	11	Lg_c	あり
f	3.1	0.6	−1.97	2b	15	Lg_f	なし
f	3.1	3.4	−1.47	2b	14	Lg_f	なし
f	3	1.2	−1.68	2b	9	Lg_f	あり
f	2.9	1	−1.65	2b	9	Lg_f	あり
f	3.3	1.1	−1.96	2b	10	Lg_c	あり
m	2.9	0.7	−1.75	2b	8	Lg_c	あり
f	3.2	0.6	−2.05	2b	10	Lg_f	あり
f	3.3	0.7	−2.09	2b	8	Lg_c	あり
m	3.3	0.9	−2.02	2b	8	Lg_f	あり
m	2.7	1.6	−1.35	3	14	Lg_c	あり
f	3.3	0.5	−2.19	2b	8	Lg_f	なし
m	2.2	1.3	−0.98	3	14	Lg_f	あり
m	3.5	1.1	−2.13	2b	10	Lg_f	あり
f	3.2	1.3	−1.83	2b	9	Lg_c	あり
f	2.7	0.6	−1.63	2b	8	Lg_c	あり
f	2.3	0.9	−1.17	3	17	Lg_c	あり
f	3	1	−1.74	2b	8	Lg_f	なし
m	2.5	1.8	−1.14	3	11	Lg_f	あり
f	3.2	0.7	−2.01	2b	8	Lg_f	なし

f	3	2.9	−1.43	2b	10	Lg_f	なし
m	2.8	1	−1.57	2b	8	Lg_c	あり
m	3.3	1.9	−1.81	2b	9	Lg_c	あり
f	2.2	3	−0.74	3	13	Lg_f	なし
m	3	1.7	−1.58	2b	8	Lg_c	あり
m	3.2	0.5	−2.1	2b	24	Lg_f	あり
m	2.7	0.6	−1.63	2b	11	Lg_c	あり
m	2.7	0.7	−1.58	2b	8	Lg_c	あり
m	3.4	1.7	−1.92	2b	8	Lg_c	あり
m	2.6	2	−1.2	3	10	Lg_c	あり
m	3.3	0.4	−2.25	2b	10	Lg_f	あり
m	3.1	1	−1.82	2b	8	Lg_f	なし
m	3.3	1.9	−1.81	2b	7	Lg_c	あり
m	2.8	2	−1.37	3	10	Lg_c	あり
m	2.4	1.6	−1.09	3	7	Lg_c	あり
f	2.2	1.2	−1	3	13	Lg_f	あり
f	2.7	5.4	−1	3	13	Lg_c	あり
f	4.2	0.5	−2.95	1	7	Lg_c	あり
f	2.2	5.8	−0.55	3	17	Lg_c	あり
m	2.2	0.5	−1.25	3	22	Lg_c	あり
f	3.8	0.4	−2.68	1	8	Lg_f	あり
m	4.2	0.6	−2.9	1	8	Lg_c	あり
m	3.5	0.5	−2.36	2a	9	Lg_c	あり
m	2.8	2	−1.37	3	9	Lg_f	あり
m	3.5	1.5	−2.05	2b	7	Lg_c	あり
m	3.2	0.8	−1.97	2b	9	Lg_f	あり
m	4	0.8	−2.65	1	8	Lg_f	あり
m	3.7	0.9	−2.36	2a	10	Lg_f	あり
m	3.7	1.2	−2.28	2a	9	Lg_f	あり
m	3	1	−1.74	2b	9	Lg_c	あり
f	2.2	2.1	−0.84	3	27	Lg_f	あり
m	2.7	2.1	−1.27	3	12	Lg_f	あり
m	2.6	0.6	−1.54	2b	11	Lg_c	あり
f	2.6	1.6	−1.26	3	15	Lg_c	あり
m	2.4	1.9	−1.04	3	14	Lg_c	あり
m	3.3	0.7	−2.09	2b	8	Lg_c	あり
m	2.8	0.7	−1.67	2b	14	Lg_c	あり
m	1.8	2.9	−0.41	3	14	Lg_c	あり
m	3.4	0.8	−2.14	2b	9	Lg_c	あり
m	3	0.5	−1.93	2b	11	Lg_f	なし
m	2.3	0.9	−1.17	3	10	Lg_c	あり
m	3.1	0.6	−1.97	2b	7	Lg_f	あり
m	2.9	0.7	−1.75	2b	7	Lg_f	あり
m	3.1	0.7	−1.92	2b	8	Lg_f	あり
f	2.5	0.8	−1.38	3	9	Lg_c	あり
f	3.4	0.8	−2.14	2b	8	Lg_c	あり
m	3	1.5	−1.62	2b	14	Lg_f	あり
f	2.8	1	−1.57	2b	10	Lg_f	あり
m	3.4	0.7	−2.18	2b	10	Lg_c	あり
f	2.5	1.3	−1.24	3	10	Lg_c	あり
f	2.7	0.9	−1.51	2b	6	Lg_c	あり
m	2.7	2.2	−1.26	3	11	Lg_f	あり
m	2.9	1.1	−1.62	2b	10	Lg_c	あり
m	2.2	1.2	−1	3	9	Lg_c	あり
f	2.3	1.4	−1.04	3	10	Lg_c	あり
m	2.5	0.7	−1.41	2b	20	Lg_f	なし

m	2.5	1.5	−1.2	3	13	Lg_f	あり
m	2.1	1.9	−0.79	3	15	Lg_c	あり
f	3.4	1.0	−2.08	2b	9	Lg_c	あり
f	3.2	0.4	−2.17	2b	8	Lg_f	なし
f	2.6	0.4	−1.66	2b	9	Lg_c	あり
m	2.8	1.7	−1.41	2b	23	Lg_c	あり
f	2.1	1.4	−0.87	3	12	Lg_c	あり
f	2.8	0.5	−1.76	2b	7	Lg_f	なし
m	3.1	0.6	−1.97	2b	11	Lg_c	あり
m	3.2	1	−1.91	2b	7	Lg_c	あり
m	2.1	19.5	−0.12	3	29	Lg_c	あり
f	2.2	1.0	−1.06	3	8	Lg_c	あり
m	3.2	1.1	−1.88	2b	11	Lg_c	あり
m	3.6	0.5	−2.44	2a	8	Lg_c	あり
m	4.0	0.7	−2.69	1	7	Lg_c	あり
m	3.1	0.5	−2.02	2b	8	Lg_c	あり
m	3.3	0.9	−2.02	2b	7	Lg_f	あり
m	2.3	1.7	−0.99	3	19	Lg_c	あり
f	2.8	0.7	−1.67	2b	9	Lg_c	あり
m	3.6	1.0	−2.25	2b	10	Lg_c	あり
f	3.4	1.4	−1.98	2b	9	Lg_c	あり
m	3.4	0.6	−2.22	2b	13.0	Lg_c	あり
m	3.2	1.6	−1.77	2b	11.0	Lg_c	あり
m	2.0	2.8	−0.59	3	11	Lg_c	あり
m	2.8	0.8	−1.63	2b	8.0	Lg_c	あり
m	3.2	1.1	−1.88	2b	9	Lg_f	あり

治療法1	治療法2	治療法3	BRTO評価	原疾患	C-P	PPIの有無	HCC
EVL	EVL	APC		Alcohol	A	なし	なし
EVL	EIS	EIS		C	A	なし	なし
CA	BRTO		成功	C	B	なし	なし
CA	なし		—	C	C	あり	なし
EIS	EIS	EIS		Alcohol	A	あり	なし
CA	EIS	EIS	—	C	B	なし	なし
EVL				C	C	あり	あり
CA	BRTO		成功	C	B	なし	なし
CA	BRTO		成功	Alcohol	B	なし	なし
CA	BRTO		成功	その他	B	なし	なし
EVL	EVL			Alcohol	C	なし	なし
EVL	EVL	EVL		C	B	なし	なし
EVL	EVL	EVL		Alcohol	B	なし	なし
CA	BRTO		成功	その他	A	なし	なし
EVL	EVL	EIS		その他	A	あり	なし
EVL	EVL	EIS		C	B	なし	あり
EVL	EVL	EIS		その他	B	なし	あり
EVL	EVL	EIS		その他	B	なし	なし
EVL	EVL	EIS		Alcohol	C	なし	なし
CA	BRTO		成功	C	B	なし	あり
EVL	EIS	EIS		その他	B	なし	なし
CA	EIS		不成功	Alcohol	C	なし	なし
EVL	EVL			C	C	なし	なし
CA	BRTO		成功	C	B	なし	なし
EVL				C	C	なし	あり
CA	BRTO		成功	C	B	あり	なし
EVL	EVL	EVL		Alcohol	B	なし	なし
EVL	EVL			C	C	なし	あり
EVL	EVL	EIS		Alcohol	B	なし	あり
EVL				C	B	なし	あり
CA	BRTO		成功	Alcohol	C	なし	なし
EVL	EVL	EVL		C	B	なし	あり
EVL	EVL			C	B	なし	あり
EVL	EVL	EVL		C	B	なし	あり
EVL	EVL	EVL		Alcohol	B	あり	なし
EVL	EVL	EVL		C	B	なし	なし
CA	BRTO		成功	C	A	なし	あり
CA	なし		—	Alcohol	C	なし	なし
CA	BRTO	その他	成功	Alcohol	C	なし	なし
CA	BRTO		成功	C	B	なし	なし
EVL	EIS			その他	A	なし	なし
EVL	EIS			Alcohol	A	なし	なし
CA	BRTO		成功	その他	B	あり	なし
EVL	EIS	EIS		C	B	あり	なし
CA	BRTO	その他	成功	その他	B	なし	なし
EVL	EVL			C	C	あり	なし
CA	BRTO		成功	C	A	あり	なし
CA	なし		—	Alcohol	C	なし	なし
CA	EIS		—	C	B	あり	あり
EVL				Alcohol	B	あり	なし
EVL	EVL	EVL		その他	B	なし	なし
EVL	EVL			C	B	あり	あり
CA	なし		—	その他	A	あり	なし
CA	O不成功でEIS追加		不成功	Alcohol	C	なし	なし
CA	BRTO		成功	Alcohol	A	なし	なし

CA	BRTO		成功	Alcohol	B	あり	あり
EVL				Alcohol	B	なし	あり
EIS	EIS	EIS		その他	B	なし	なし
CA	なし		—	その他	B	あり	なし
EVL	EVL			Alcohol	B	なし	なし
CA	BRTO		成功	C	B	なし	なし
EVL	EIS	EIS		C	B	あり	あり
EIS	EIS	EIS		C	B	なし	なし
EIS	EIS	EIS		その他	A	なし	なし
EVL	EVL			Alcohol	C	なし	あり
CA	BRTO		成功	その他	A	なし	なし
CA	BRTO		成功	Alcohol	B	なし	なし
EVL	EIS	EIS		Alcohol	B	なし	なし
EVL	EIS	EIS		Alcohol	B	なし	なし
EVL	EVL			Alcohol	C	なし	なし
CA	なし		—	C	C	なし	あり
EVL				Alcohol	C	なし	あり
EVL	EVL	EIS		Alcohol	A	あり	なし
EVL				C	C	あり	あり
EVL				C	C	あり	なし
CA	なし		—	その他	B	なし	なし
EVL				Alcohol	B	なし	なし
EIS	EIS			Alcohol	A	あり	なし
CA	BRTO		成功	Alcohol	B	なし	なし
EVL	EIS			Alcohol	B	あり	なし
CA	なし		—	Alcohol	B	なし	あり
CA	BRTO		成功	その他	A	なし	なし
CA	なし		—	その他	A	なし	あり
CA	BRTO		成功	Alcohol	B	なし	なし
EVL	EVL			C	B	なし	あり
CA	なし		—	C	C	なし	なし
CA	なし		—	Alcohol	C	なし	なし
EVL				C	B	あり	あり
EVL	EIS			Alcohol	B	なし	あり
EVL				C	C	あり	あり
EVL	EIS			C	A	あり	あり
EVL	EVL			Alcohol	B	なし	なし
EVL				Alcohol	C	なし	なし
EVL	EIS	EIS		C	B	なし	あり
CA	なし		—	C	A	なし	なし
EVL	EIS			C	B	なし	なし
CA	BRTO		成功	C	A	なし	なし
CA	なし		—	Alcohol	B	なし	なし
CA	BRTO		成功	C	A	なし	なし
EVL	EIS	EIS		C	B	なし	なし
EVL	EIS	EIS		その他	B	なし	なし
CA	EIS		—	その他	B	なし	なし
CA	なし		—	C	B	なし	なし
EVL	EIS			その他	C	あり	なし
EVL				その他	B	なし	なし
EVL	EIS			その他	B	なし	なし
CA	O不成功でEIS追加		不成功	その他	B	なし	なし
EVL	EVL			その他	C	なし	なし
EVL	EIS	EIS		その他	C	あり	なし
EVL				その他	C	なし	あり
CA	BRTO		成功	C	B	なし	なし

CA	BRTO		成功	その他	B	なし	なし
EVL	EIS	EIS		その他	C	なし	なし
EVL	EIS	EIS		その他	B	なし	なし
CA	なし		—	C	B	なし	あり
EVL	EIS	EIS		C	B	なし	なし
EVL				その他	B	なし	なし
EVL	EIS	EIS		C	C	なし	なし
CA	BRTO		成功	その他	A	なし	なし
EVL	EIS			その他	肝硬変なし	あり	なし
EVL				その他	B	あり	なし
EVL	EVL			B	C	あり	あり
EVL				C	B	なし	なし
EIS	EIS	EIS		B	A	なし	なし
EVL	EVL			Alcohol	B	なし	なし
EVL	EIS			その他	A	あり	なし
EVL	EVL			Alcohol	A	あり	あり
CA	BRTO		成功	その他	B	なし	なし
EVL				C	B	なし	なし
EVL	EIS	EIS		その他	B	なし	なし
EVL	EIS	EIS		Alcohol	B	なし	なし
EVL	EIS			Alcohol	B	なし	なし
EVL	EIS			Alcohol	B	あり	なし
EVL	EIS	EIS		Alcohol	B	なし	なし
EVL				Alcohol	C	なし	なし
EVL	EVL	EVL		Alcohol	B	なし	あり
CA	EIS		不成功	Alcohol	B	あり	なし

PVT(T)	再発	転帰	生存打ち切り	生存時間	出血時間
なし	あり	不明	打ち切り	115.66	115.66
なし	あり	死亡	非打ち切り	97.86	97.86
なし	あり	不明	打ち切り	114.63	114.63
なし	なし	在院死亡	非打ち切り	0.86	0.86
なし	あり	不明	打ち切り	82.30	82.30
なし	不明	不明	打ち切り	76.16	76.16
あり	なし	在院死亡	非打ち切り	0.00	0.00
なし	なし(食道)	死亡	非打ち切り	48.76	48.76
なし	なし	死亡	非打ち切り	59.26	59.26
なし	あり	不明	打ち切り	13.26	13.26
なし	あり	死亡	非打ち切り	59.63	15.00
なし	あり	生存	打ち切り	166.79	166.79
なし	あり	死亡	非打ち切り	137.39	33.99
なし	なし(食道)	不明	打ち切り	94.16	94.16
なし	あり	不明	打ち切り	59.07	59.07
なし	あり	不明	打ち切り	24.33	24.33
あり	あり	死亡	非打ち切り	6.36	3.30
なし	あり	不明	打ち切り	130.10	130.10
なし	不明	不明	打ち切り	0.53	0.53
なし	不明	不明	打ち切り	3.43	3.43
なし	なし	死亡	非打ち切り	3.53	3.53
なし	なし	不明	打ち切り	56.49	56.49
なし	不明	不明	打ち切り	1.63	1.63
なし	不明	不明	打ち切り	129.07	129.07
あり	なし	在院死亡	非打ち切り	0.00	0.00
なし	なし	生存	打ち切り	147.49	147.49
なし	あり	死亡	非打ち切り	6.26	3.76
なし	不明	不明	打ち切り	0.92	0.92
なし	なし	死亡	非打ち切り	55.76	55.76
なし	あり	死亡	非打ち切り	7.89	6.89
なし	不明	不明	打ち切り	104.69	104.69
なし	あり	生存	打ち切り	139.99	139.99
なし	あり	不明	打ち切り	25.76	25.76
なし	なし	死亡	非打ち切り	1.49	1.49
なし	あり	生存	打ち切り	137.66	9.43
なし	不明	不明	打ち切り	11.39	11.39
なし	なし	不明	打ち切り	1.13	1.13
なし	不明	不明	打ち切り	3.66	3.66
なし	不明	不明	打ち切り	48.66	48.66
なし	不明	不明	打ち切り	0.82	0.82
なし	不明	不明	打ち切り	13.89	13.89
なし	あり	死亡	非打ち切り	114.46	67.72
なし	なし	生存	打ち切り	126.43	126.43
なし	あり	生存	打ち切り	126.16	126.16
なし	なし(食道)	生存	打ち切り	126.13	30.33
なし	なし	死亡	非打ち切り	8.92	8.92
なし	なし(食道)	生存	打ち切り	124.89	124.89
なし	なし	死亡	非打ち切り	25.72	25.72
なし	不明	不明	打ち切り	37.00	37.00
なし	あり	不明	打ち切り	59.53	10.56
なし	不明	不明	打ち切り	12.00	12.00
なし	なし	在院死亡	非打ち切り	0.49	0.49
なし	あり	死亡	非打ち切り	65.66	65.66
なし	不明	不明	打ち切り	0.66	0.66
なし	不明	不明	打ち切り	28.69	28.69

なし	不明	不明	打ち切り	43.56	43.56
あり	不明	不明	打ち切り	3.66	3.66
なし	不明	不明	打ち切り	0.86	0.86
なし	不明	不明	打ち切り	0.13	0.13
なし	あり	不明	打ち切り	10.79	10.39
なし	不明	不明	打ち切り	12.20	12.20
なし	あり	不明	打ち切り	34.66	34.66
なし	不明	不明	打ち切り	0.76	0.76
なし	あり	生存	打ち切り	103.76	103.76
なし	なし	死亡	非打ち切り	60.10	60.10
なし	不明	死亡	非打ち切り	59.53	59.53
なし	なし	死亡	非打ち切り	74.33	74.33
なし	あり	生存	打ち切り	100.69	100.69
なし	あり	不明	打ち切り	7.92	4.07
なし	あり	死亡	非打ち切り	16.92	16.89
なし	なし(食道)	死亡	非打ち切り	13.86	13.86
あり	なし	在院死亡	非打ち切り	0.03	0.03
なし	あり	生存	打ち切り	92.00	26.13
あり	なし	在院死亡	非打ち切り	1.56	1.56
なし	なし	死亡	非打ち切り	19.59	19.59
なし	不明	不明	打ち切り	0.30	0.30
あり	なし	不明	打ち切り	77.66	77.66
なし	あり	不明	打ち切り	49.07	4.89
なし	なし	不明	打ち切り	23.46	23.46
なし	あり	死亡	非打ち切り	64.86	12.00
なし	なし	死亡	非打ち切り	6.43	6.43
なし	なし	不明	打ち切り	12.63	12.63
なし	なし	生存	打ち切り	77.86	77.86
なし	なし(食道)	死亡	非打ち切り	45.23	22.95
あり	あり	死亡	非打ち切り	1.53	1.46
なし	なし	在院死亡	非打ち切り	1.49	1.49
あり	なし(食道)	在院死亡	非打ち切り	1.82	0.92
あり	なし	在院死亡	非打ち切り	1.16	1.16
なし	なし	生存	打ち切り	65.43	65.43
あり	あり	不明	打ち切り	2.39	1.46
なし	不明	不明	打ち切り	14.30	14.30
なし	不明	不明	打ち切り	2.92	2.92
なし	不明	不明	打ち切り	0.46	0.46
なし	不明	不明	打ち切り	30.46	30.46
なし	なし	死亡	非打ち切り	22.20	22.20
なし	あり	死亡	非打ち切り	31.95	29.66
なし	なし	不明	打ち切り	34.30	34.30
なし	なし	生存	打ち切り	52.23	52.23
なし	なし	生存	打ち切り	51.43	51.43
なし	あり	生存	打ち切り	50.26	50.26
なし	あり	生存	打ち切り	50.20	50.20
なし	不明	不明	打ち切り	1.86	1.86
なし	なし	生存	打ち切り	49.92	49.92
なし	不明	不明	打ち切り	20.56	20.56
なし	なし	不明	打ち切り	31.16	31.16
なし	なし	不明	打ち切り	39.59	39.59
なし	なし(Lg-c)	死亡	非打ち切り	24.26	24.26
なし	あり	死亡	非打ち切り	22.72	22.72
なし	なし	不明	打ち切り	12.00	12.00
なし	なし	不明	打ち切り	37.92	37.92
なし	なし	生存	打ち切り	37.92	37.92

なし	なし	死亡	非打ち切り	24.13	24.13
なし	なし	生存	打ち切り	35.26	35.26
なし	あり	生存	打ち切り	34.76	34.76
なし	なし	不明	打ち切り	6.36	6.36
なし	なし	生存	打ち切り	32.95	32.95
なし	なし	不明	打ち切り	1.69	1.69
なし	なし	死亡	非打ち切り	5.23	5.23
なし	なし	生存	打ち切り	28.46	28.46
あり	あり	生存	打ち切り	25.46	25.46
なし	なし	不明	打ち切り	18.72	18.72
あり	あり	在院死亡	非打ち切り	0.43	0.03
なし	なし	生存	打ち切り	21.66	21.66
あり	なし	生存	打ち切り	19.82	19.82
なし	あり	生存	打ち切り	19.20	19.20
なし	あり	生存	打ち切り	16.76	16.76
あり	あり	生存	打ち切り	16.23	16.23
なし	不明	不明	打ち切り	3.26	3.26
なし	なし	在院死亡	非打ち切り	0.36	0.36
なし	あり	生存	打ち切り	15.86	15.86
なし	あり	生存	打ち切り	13.36	13.36
なし	なし	生存	打ち切り	10.07	10.07
なし	あり	生存	打ち切り	9.07	9.07
なし	なし	生存	打ち切り	4.63	4.63
なし	不明	生存	打ち切り	4.63	4.63
あり	なし	生存	打ち切り	2.33	2.33
なし	なし	生存	打ち切り	2.30	2.30
