# Supplementary material for: Long-term outcomes of patients with cirrhosis presenting with bleeding gastric varices
Source: PLoS One. 2022 Mar 15;17(3):e0264359. doi: 10.1371/journal.pone.0264359 (PMC8923466; doi:10.1371/journal.pone.0264359)
Supplement: S2 File — (PDF) [file pone.0264359.s002.pdf]

Sex	Site of GV	Etiology	Albumin	T-bil	ALBI score	ALBI grade	MELD
m	GOV	Alcohol	3.4	0.4	−2.34	2a	7
m	GOV	HCV	3.9	1	−2.5	2a	10
f	FV	HCV	3.2	1.1	−1.88	2b	9
f	FV	HCV	2.4	3.1	−0.9	3	16
m	GOV	Alcohol	4.2	1	−2.76	1	9
f	FV	HCV	3.1	1.5	−1.71	2b	9
m	GOV	HCV	2.4	2.1	−1.01	3	15
f	FV	HCV	3.8	1.3	−2.34	2a	13
m	FV	Alcohol	2.6	1.3	−1.32	3	9
f	FV	Other	3.7	1	−2.33	2a	9
m	GOV	Alcohol	2.9	1.1	−1.62	2b	9
f	GOV	HCV	3.2	1	−1.91	2b	11
m	GOV	Alcohol	3.7	1.5	−2.22	2b	9
f	FV	Other	3.4	1.3	−2	2b	8
m	GOV	Other	4	0.5	−2.78	1	8
m	GOV	HCV	3	1	−1.74	2b	9
m	GOV	Other	4.4	0.5	−3.12	1	7
f	GOV	Other	3.4	0.5	−2.27	2b	6
f	GOV	Alcohol	3.5	2.9	−1.86	2b	8
m	FV	HCV	2.8	2.1	−1.35	3	10
m	GOV	Other	2.7	1.2	−1.43	2b	20
m	FV	Alcohol	2.8	1.2	−1.51	2b	13
m	GOV	HCV	2.5	1.1	−1.28	3	22
m	FV	HCV	3	1.7	−1.58	2b	10
m	GOV	HCV	2.6	1.9	−1.21	3	24
m	FV	HCV	3.7	1.1	−2.3	2a	18
f	GOV	Alcohol	3.1	0.9	−1.85	2b	11
f	GOV	HCV	2.4	3.8	−0.84	3	11
m	GOV	Alcohol	3.4	2.4	−1.83	2b	16
m	GOV	HCV	2.9	1.2	−1.6	2b	20
m	FV	Alcohol	3.3	0.8	−2.06	2b	7
f	GOV	HCV	3.5	0.7	−2.26	2b	9
f	GOV	HCV	2.8	0.4	−1.83	2b	7
m	GOV	HCV	3	0.9	−1.77	2b	9
m	GOV	Alcohol	3.1	1.2	−1.77	2b	11
m	GOV	HCV	2.7	0.7	−1.58	2b	11
f	FV	HCV	3.1	0.6	−1.97	2b	15
f	FV	Alcohol	3.1	3.4	−1.47	2b	14
f	FV	Alcohol	3	1.2	−1.68	2b	9
f	FV	HCV	2.9	1	−1.65	2b	9
f	GOV	Other	3.3	1.1	−1.96	2b	10
m	GOV	Alcohol	2.9	0.7	−1.75	2b	8
f	FV	Other	3.2	0.6	−2.05	2b	10
f	GOV	HCV	3.3	0.7	−2.09	2b	8
m	FV	Other	3.3	0.9	−2.02	2b	8
m	GOV	HCV	2.7	1.6	−1.35	3	14
f	FV	HCV	3.3	0.5	−2.19	2b	8
m	FV	Alcohol	2.2	1.3	−0.98	3	14
m	FV	HCV	3.5	1.1	−2.13	2b	10
f	GOV	Alcohol	3.2	1.3	−1.83	2b	9
f	GOV	Other	2.7	0.6	−1.63	2b	8
f	GOV	HCV	2.3	0.9	−1.17	3	17
f	FV	Other	3	1	−1.74	2b	8
m	FV	Alcohol	2.5	1.8	−1.14	3	11
f	FV	Alcohol	3.2	0.7	−2.01	2b	8

f	FV	Alcohol	3	2.9	−1.43	2b	10
m	GOV	Alcohol	2.8	1	−1.57	2b	8
m	GOV	Other	3.3	1.9	−1.81	2b	9
f	FV	Other	2.2	3	−0.74	3	13
m	GOV	Alcohol	3	1.7	−1.58	2b	8
m	FV	HCV	3.2	0.5	−2.1	2b	24
m	GOV	HCV	2.7	0.6	−1.63	2b	11
m	GOV	HCV	2.7	0.7	−1.58	2b	8
m	GOV	Other	3.4	1.7	−1.92	2b	8
m	GOV	Alcohol	2.6	2	−1.2	3	10
m	FV	Other	3.3	0.4	−2.25	2b	10
m	FV	Alcohol	3.1	1	−1.82	2b	8
m	GOV	Alcohol	3.3	1.9	−1.81	2b	7
m	GOV	Alcohol	2.8	2	−1.37	3	10
m	GOV	Alcohol	2.4	1.6	−1.09	3	7
f	FV	HCV	2.2	1.2	−1	3	13
f	GOV	Alcohol	2.7	5.4	−1	3	13
f	GOV	Alcohol	4.2	0.5	−2.95	1	7
f	GOV	HCV	2.2	5.8	−0.55	3	17
m	GOV	HCV	2.2	0.5	−1.25	3	22
f	FV	Other	3.8	0.4	−2.68	1	8
m	GOV	Alcohol	4.2	0.6	−2.9	1	8
m	GOV	Alcohol	3.5	0.5	−2.36	2a	9
m	FV	Alcohol	2.8	2	−1.37	3	9
m	GOV	Alcohol	3.5	1.5	−2.05	2b	7
m	FV	Alcohol	3.2	0.8	−1.97	2b	9
m	FV	Other	4	0.8	−2.65	1	8
m	FV	Other	3.7	0.9	−2.36	2a	10
m	FV	Alcohol	3.7	1.2	−2.28	2a	9
m	GOV	HCV	3	1	−1.74	2b	9
f	FV	HCV	2.2	2.1	−0.84	3	27
m	FV	Alcohol	2.7	2.1	−1.27	3	12
m	GOV	HCV	2.6	0.6	−1.54	2b	11
f	GOV	Alcohol	2.6	1.6	−1.26	3	15
m	GOV	HCV	2.4	1.9	−1.04	3	14
m	GOV	HCV	3.3	0.7	−2.09	2b	8
m	GOV	Alcohol	2.8	0.7	−1.67	2b	14
m	GOV	Alcohol	1.8	2.9	−0.41	3	14
m	GOV	HCV	3.4	0.8	−2.14	2b	9
m	FV	HCV	3	0.5	−1.93	2b	11
m	GOV	HCV	2.3	0.9	−1.17	3	10
m	FV	HCV	3.1	0.6	−1.97	2b	7
m	FV	Alcohol	2.9	0.7	−1.75	2b	7
m	FV	HCV	3.1	0.7	−1.92	2b	8
f	GOV	HCV	2.5	0.8	−1.38	3	9
f	GOV	Other	3.4	0.8	−2.14	2b	8
m	FV	Other	3	1.5	−1.62	2b	14
f	FV	HCV	2.8	1	−1.57	2b	10
m	GOV	Other	3.4	0.7	−2.18	2b	10
f	GOV	Other	2.5	1.3	−1.24	3	10
f	GOV	Other	2.7	0.9	−1.51	2b	6
m	FV	Other	2.7	2.2	−1.26	3	11
m	GOV	Other	2.9	1.1	−1.62	2b	10
m	GOV	Other	2.2	1.2	−1	3	9
f	GOV	Other	2.3	1.4	−1.04	3	10
m	FV	HCV	2.5	0.7	−1.41	2b	20

m	FV	Other	2.5	1.5	−1.2	3	13
m	GOV	Other	2.1	1.9	−0.79	3	15
f	GOV	Other	3.4	1.0	−2.08	2b	9
f	FV	HCV	3.2	0.4	−2.17	2b	8
f	GOV	HCV	2.6	0.4	−1.66	2b	9
m	GOV	Other	2.8	1.7	−1.41	2b	23
f	GOV	HCV	2.1	1.4	−0.87	3	12
f	FV	Other	2.8	0.5	−1.76	2b	7
m	GOV	Other	3.1	0.6	−1.97	2b	11
m	GOV	Other	3.2	1	−1.91	2b	7
m	GOV	HBV	2.1	19.5	−0.12	3	29
f	GOV	HCV	2.2	1.0	−1.06	3	8
m	GOV	HBV	3.2	1.1	−1.88	2b	11
m	GOV	Alcohol	3.6	0.5	−2.44	2a	8
m	GOV	Other	4.0	0.7	−2.69	1	7
m	GOV	Alcohol	3.1	0.5	−2.02	2b	8
m	FV	Other	3.3	0.9	−2.02	2b	7
m	GOV	HCV	2.3	1.7	−0.99	3	19
f	GOV	Other	2.8	0.7	−1.67	2b	9
m	GOV	Alcohol	3.6	1.0	−2.25	2b	10
f	GOV	Alcohol	3.4	1.4	−1.98	2b	9
m	GOV	Alcohol	3.4	0.6	−2.22	2b	13.0
m	GOV	Alcohol	3.2	1.6	−1.77	2b	11.0
m	GOV	Alcohol	2.0	2.8	−0.59	3	11
m	GOV	Alcohol	2.8	0.8	−1.63	2b	8.0
m	FV	Alcohol	3.2	1.1	−1.88	2b	9

CTP grade	Initial Tx	Additional Tx 1	Additional Tx 2	BRTO	PPI	EV	HCC
A	EVL	EVL	APC		absent	present	absent
A	EVL	EIS	EIS		absent	present	absent
B	EIS(CA)	BRTO		success	absent	present	absent
C	EIS(CA)			—	present	present	absent
A	EIS	EIS	EIS		present	present	absent
B	EIS(CA)	EIS	EIS	—	absent	present	absent
C	EVL				present	present	present
B	EIS(CA)	BRTO		success	absent	present	absent
B	EIS(CA)	BRTO		success	absent	present	absent
B	EIS(CA)	BRTO		success	absent	absent	absent
C	EVL	EVL			absent	present	absent
B	EVL	EVL	EVL		absent	present	absent
B	EVL	EVL	EVL		absent	present	absent
A	EIS(CA)	BRTO		success	absent	absent	absent
A	EVL	EVL	EIS		present	present	absent
B	EVL	EVL	EIS		absent	present	present
B	EVL	EVL	EIS		absent	present	present
B	EVL	EVL	EIS		absent	present	absent
C	EVL	EVL	EIS		absent	present	absent
B	EIS(CA)	BRTO		success	absent	absent	present
B	EVL	EIS	EIS		absent	present	absent
C	EIS(CA)	EIS		failure	absent	absent	absent
C	EVL	EVL			absent	present	absent
B	EIS(CA)	BRTO		success	absent	absent	absent
C	EVL				absent	present	present
B	EIS(CA)	BRTO		success	present	absent	absent
B	EVL	EVL	EVL		absent	present	absent
C	EVL	EVL			absent	present	present
B	EVL	EVL	EIS		absent	present	present
B	EVL				absent	present	present
C	EIS(CA)	BRTO		success	absent	absent	absent
B	EVL	EVL	EVL		absent	present	present
B	EVL	EVL			absent	present	present
B	EVL	EVL	EVL		absent	present	present
B	EVL	EVL	EVL		present	present	absent
B	EVL	EVL	EVL		absent	present	absent
A	EIS(CA)	BRTO		success	absent	absent	present
C	EIS(CA)	なし		—	absent	absent	absent
C	EIS(CA)	BRTO	other	success	absent	present	absent
B	EIS(CA)	BRTO		success	absent	present	absent
A	EVL	EIS			absent	present	absent
A	EVL	EIS			absent	present	absent
B	EIS(CA)	BRTO		success	present	present	absent
B	EVL	EIS	EIS		present	present	absent
B	EIS(CA)	BRTO	other	success	absent	present	absent
C	EVL	EVL			present	present	absent
A	EIS(CA)	BRTO		success	present	absent	absent
C	EIS(CA)			—	absent	present	absent
B	EIS(CA)	EIS		—	present	present	present
B	EVL				present	present	absent
B	EVL	EVL	EVL		absent	present	absent
B	EVL	EVL			present	present	present
A	EIS(CA)			—	present	absent	absent
C	EIS(CA)	EIS		failure	absent	present	absent
A	EIS(CA)	BRTO		success	absent	absent	absent

B	EIS(CA)	BRTO		success	present	absent	present
B	EVL				absent	present	present
B	EIS	EIS	EIS		absent	present	absent
B	EIS(CA)			—	present	absent	absent
B	EVL	EVL			absent	present	absent
B	EIS(CA)	BRTO		success	absent	present	absent
B	EVL	EIS	EIS		present	present	present
B	EIS	EIS	EIS		absent	present	absent
A	EIS	EIS	EIS		absent	present	absent
C	EVL	EVL			absent	present	present
A	EIS(CA)	BRTO		success	absent	present	absent
B	EIS(CA)	BRTO		success	absent	absent	absent
B	EVL	EIS	EIS		absent	present	absent
B	EVL	EIS	EIS		absent	present	absent
C	EVL	EVL			absent	present	absent
C	EIS(CA)			—	absent	present	present
C	EVL				absent	present	present
A	EVL	EVL	EIS		present	present	absent
C	EVL				present	present	present
C	EVL				present	present	absent
B	EIS(CA)			—	absent	present	absent
B	EVL				absent	present	absent
A	EIS	EIS			present	present	absent
B	EIS(CA)	BRTO		success	absent	present	absent
B	EVL	EIS			present	present	absent
B	EIS(CA)			—	absent	present	present
A	EIS(CA)	BRTO		success	absent	present	absent
A	EIS(CA)			—	absent	present	present
B	EIS(CA)	BRTO		success	absent	present	absent
B	EVL	EVL			absent	present	present
C	EIS(CA)			—	absent	present	absent
C	EIS(CA)			—	absent	present	absent
B	EVL				present	present	present
B	EVL	EIS			absent	present	present
C	EVL				present	present	present
A	EVL	EIS			present	present	present
B	EVL	EVL			absent	present	absent
C	EVL				absent	present	absent
B	EVL	EIS	EIS		absent	present	present
A	EIS(CA)			—	absent	absent	absent
B	EVL	EIS			absent	present	absent
A	EIS(CA)	BRTO		success	absent	present	absent
B	EIS(CA)			—	absent	present	absent
A	EIS(CA)	BRTO		success	absent	present	absent
B	EVL	EIS	EIS		absent	present	absent
B	EVL	EIS	EIS		absent	present	absent
B	EIS(CA)	EIS		—	absent	present	absent
B	EIS(CA)	なし		—	absent	present	absent
C	EVL	EIS			present	present	absent
B	EVL				absent	present	absent
B	EVL	EIS			absent	present	absent
B	EIS(CA)	EIS		failure	absent	present	absent
C	EVL	EVL			absent	present	absent
C	EVL	EIS	EIS		present	present	absent
C	EVL				absent	present	present
B	EIS(CA)	BRTO		success	absent	absent	absent

B	EIS(CA)	BRTO		success	absent	present	absent
C	EVL	EIS	EIS		absent	present	absent
B	EVL	EIS	EIS		absent	present	absent
B	EIS(CA)			—	absent	absent	present
B	EVL	EIS	EIS		absent	present	absent
B	EVL				absent	present	absent
C	EVL	EIS	EIS		absent	present	absent
A	EIS(CA)	BRTO		success	absent	absent	absent
N/A	EVL	EIS			present	present	absent
B	EVL				present	present	absent
C	EVL	EVL			present	present	present
B	EVL				absent	present	absent
A	EIS	EIS	EIS		absent	present	absent
B	EVL	EVL			absent	present	absent
A	EVL	EIS			present	present	absent
A	EVL	EVL			present	present	present
B	EIS(CA)	BRTO		success	absent	present	absent
B	EVL				absent	present	absent
B	EVL	EIS	EIS		absent	present	absent
B	EVL	EIS	EIS		absent	present	absent
B	EVL	EIS			absent	present	absent
B	EVL	EIS			present	present	absent
B	EVL	EIS	EIS		absent	present	absent
C	EVL				absent	present	absent
B	EVL	EVL	EVL		absent	present	present
B	EIS(CA)	EIS		failure	present	present	absent

PVT(T)	Rebleeding	Outcome	Survival time
absent	absent	unknown	115.66
absent	absent	death	97.86
absent	absent	unknown	114.63
absent	absent	death	0.86
absent	absent	unknown	82.30
absent	absent	unknown	76.16
present	absent	death	0.00
absent	absent	death	48.76
absent	absent	death	59.26
absent	absent	unknown	13.26
absent	present	death	59.63
absent	absent	survival	166.79
absent	present	death	137.39
absent	absent	unknown	94.16
absent	absent	unknown	59.07
absent	absent	unknown	24.33
present	present	death	6.36
absent	absent	unknown	130.10
absent	absent	unknown	0.53
absent	absent	unknown	3.43
absent	absent	death	3.53
absent	absent	unknown	56.49
absent	absent	unknown	1.63
absent	absent	unknown	129.07
present	absent	death	0.00
absent	absent	survival	147.49
absent	present	death	6.26
absent	absent	unknown	0.92
absent	absent	death	55.76
absent	present	death	7.89
absent	absent	unknown	104.69
absent	absent	survival	139.99
absent	absent	unknown	25.76
absent	absent	death	1.49
absent	present	survival	137.66
absent	absent	unknown	11.39
absent	absent	unknown	1.13
absent	absent	unknown	3.66
absent	absent	unknown	48.66
absent	absent	unknown	0.82
absent	absent	unknown	13.89
absent	present	death	114.46
absent	absent	survival	126.43
absent	absent	survival	126.16
absent	present	survival	126.13
absent	absent	death	8.92
absent	absent	survival	124.89
absent	absent	death	25.72
absent	absent	unknown	37.00
absent	present	unknown	59.53
absent	absent	unknown	12.00
absent	absent	death	0.49
absent	absent	death	65.66
absent	absent	unknown	0.66
absent	absent	unknown	28.69

absent	absent	unknown	43.56
present	absent	unknown	3.66
absent	absent	unknown	0.86
absent	absent	unknown	0.13
absent	present	unknown	10.79
absent	absent	unknown	12.20
absent	absent	unknown	34.66
absent	absent	unknown	0.76
absent	absent	survival	103.76
absent	absent	death	60.10
absent	absent	death	59.53
absent	absent	death	74.33
absent	absent	survival	100.69
absent	present	unknown	7.92
absent	present	death	16.92
absent	absent	death	13.86
present	absent	death	0.03
absent	present	survival	92.00
present	absent	death	1.56
absent	absent	death	19.59
absent	absent	unknown	0.30
present	absent	unknown	77.66
absent	present	unknown	49.07
absent	absent	unknown	23.46
absent	present	death	64.86
absent	absent	death	6.43
absent	absent	unknown	12.63
absent	absent	survival	77.86
absent	present	death	45.23
present	present	death	1.53
absent	absent	death	1.49
present	present	death	1.82
present	absent	death	1.16
absent	absent	survival	65.43
present	present	unknown	2.39
absent	absent	unknown	14.30
absent	absent	unknown	2.92
absent	absent	unknown	0.46
absent	absent	unknown	30.46
absent	absent	death	22.20
absent	present	death	31.95
absent	absent	unknown	34.30
absent	absent	survival	52.23
absent	absent	survival	51.43
absent	absent	survival	50.26
absent	absent	survival	50.20
absent	absent	unknown	1.86
absent	absent	survival	49.92
absent	absent	unknown	20.56
absent	absent	unknown	31.16
absent	absent	unknown	39.59
absent	present	death	24.26
absent	absent	death	22.72
absent	absent	unknown	12.00
absent	absent	unknown	37.92
absent	absent	survival	37.92

absent	absent	death	24.13
absent	absent	survival	35.26
absent	absent	survival	34.76
absent	absent	unknown	6.36
absent	absent	survival	32.95
absent	absent	unknown	1.69
absent	absent	death	5.23
absent	absent	survival	28.46
present	absent	survival	25.46
absent	absent	unknown	18.72
present	present	death	0.43
absent	absent	survival	21.66
present	absent	survival	19.82
absent	absent	survival	19.20
absent	absent	survival	16.76
present	absent	survival	16.23
absent	absent	unknown	3.26
absent	absent	death	0.36
absent	absent	survival	15.86
absent	absent	survival	13.36
absent	absent	survival	10.07
absent	absent	survival	9.07
absent	absent	survival	4.63
absent	absent	survival	4.63
present	absent	survival	2.33
absent	absent	survival	2.30
